# Polygenic and multi locus heritability of alcoholism: Novel therapeutic targets to overcome psychological deficits

**DOI:** 10.15761/JSIN.1000240

**Published:** 2020-11-12

**Authors:** Kenneth Blum, David Baron, Rehan Jalali, Edward J Modestino, Bruce Steinberg, Igor Elman, Rajendra D Badgaiyan, Mark S Gold

**Affiliations:** 1 Western University Health Sciences, Pomona, CA, USA; 2 Institute of Psychology, ELTE Eotvos Lorand University, Budapest, Hungary; 3 Division of Nutrigenomics, Genomic Testing Center Geneus Health, LLC, San Antonio, TX, USA; 4 Department of Psychiatry, University of Vermont, VT, USA; 5 Department of Psychiatry, Wright University Boonshoff School of Medicine, Dayton, OH., USA; 6 The Kenneth Blum Behavioral Neurogenetic Institute (Division of iVitalize Inc.), Austin, Tx, USA; 7 Department of Psychology, Curry College, Milton, MA, USA; 8 Department of Psychiatry, Harvard School of Medicine, Cambridge, MA, USA; 9 Department of Psychiatry, Icahn School of Medicine at Mount Sinai, New York, NY, USA; 10 Department of Psychiatry, South Texas Veteran Health Care System, Audie L. Murphy I Memorial VA Hospital, San Antonio, TX. and Long School of Medicine, University of Texas Medical Center, San Antonio TX, USA; 11 Department of Psychiatry, Washington University School of Medicine, St. Louis, Mo. USA

**Keywords:** alcoholism, alcohol use disorder (AUD), dopamine, hypodopaminergia, multi-locus, genetic addiction risk system (GARS), reward deficiency syndrome (RDS), anti -reward symptomatology

Results of a recent study from our laboratory, along with Hungarian scientists suggest a large overlap between the occurrence of substance and non-substance addictions and behaviors and underlies the importance of investigating the possible common psychological, genetic and neural pathways. These data further support concepts such as the Reward Deficiency Syndrome and anti- reward symptomatology and the component model of addictions that propose a common phenomenological and etiological background of different addictive and related behaviors. Alcoholism is a very complex trait with epigenetic impact. However, we have argued that a number of candidate genes that interact in the brain circuitry involving the established Brain Reward Cascade provides an acceptable predictive blue -print as generated by the Genetic Addiction Risk System (GARS) consisting of multi- polygenic loci. Importantly, alcohol use disorder (AUD) represents a major and ongoing public health concern with 12-month prevalence estimates of 5.6% in the United States. Quantitative genetic studies suggest a heritability of approximately 50% for AUD, and as a result, significant efforts have been made to identify specific variation within the genome related to the etiology of AUD especially in our young population. Recent results and known psychological deficits based even on a few dopaminergic genetic polymorphisms persuasively suggest that the classic view of D1-D2 functional antagonism does not hold true for all dimensions of reward-related RDS behaviors, and that D2 neurons play a more prominent pro-motivation role as we argued previously. Moreover, NIAAA extensive investigation revealed the importance of complex signaling pathways in identifying factors responsible for complex traits such as alcohol consumption. We the authors hereby emphasize the importance of genetic antecedents of alcohol consumption that loads onto many psychological deficits. The take home message is that the brain physiological impairment does not actually display a specific alcohol disorder and this is further based on no known particular alcohol receptor per se. However, understanding the gene X environment interplay in terms of brain reward processing seems most prudent especially in the face of the stressful COVID 19 pandemic. Precision translational therapeutics derived from these doctrines, may help victims of RDS to dig themselves out of a “hypodopaminergic ditch”.

Our previous work [1] along with others show that in experimental neurobiological trials when the participants expected a reward, activation of the nucleus accumbens (NAc) appeared to moderately heritable, as were scores for physical anhedonia and pleasure [2]. Moreover, NAc activation and physical anhedonia scores appeared to be influenced by shared genes; physical anhedonia and pleasure also appeared to share some of the same genes [3]. In fact, the results of a recent study from our laboratory, along with Hungarian scientists suggest a large overlap between the occurrence of substance and non-substance addictions and behaviors and underlies the importance of investigating the possible common psychological, genetic and neural pathways. These data further support concepts such as the Reward Deficiency Syndrome and the component model of addictions that propose a common phenomenological and etiological background of different addictive and related behaviors [4,5].

Dopamine is a major component in the mechanisms involving brain function [6]. The dopaminergic system plays important roles in neuromodulation, such as motor control, motivation, reward, cognitive function, maternal, and reproductive behaviors. It is well known that certain polymorphisms of a number of reward genes, including the dopamine D2 receptor gene, play a role in the function of dopamine [7]. Moreover, alcoholism results from a dysfunction in the mesolimbic system of the brain, which directly links abnormal craving behavior with a defect in the Dopamine D2 Receptor Gene (DRD2) as well as other dopaminergic genes (D1, D3, D4, and D5, DATA1, MAO, COMT), including many genes associated with the brain reward function [8–10].

The roles of specific candidate genes have been the subject of much debate and to date there is no consensus regarding a unique gene panel for alcohol addiction. There are many candidate genes representing the neurochemical mechanisms involved in reward dependence behaviors linked to mesolimbic circuitry [11]. Importantly, alcohol use disorder (AUD) represents a major and ongoing public health concern with 12-month prevalence estimates of 5.6% in the United States. Quantitative genetic studies suggest a heritability of approximately 50% for AUD, and as a result, significant efforts have been made to identify specific variation within the genome related to the etiology of AUD especially in our young population [12]. Given the limited number of replicable findings that have emerged from genome-wide linkage and candidate gene association studies, more recent efforts have focused on the use of genome-wide association studies (GWAS). These studies have suggested that hundreds of variants across the genome, most of small effect (R^2^ < 0.002), contribute to the genetic etiology of AUD [13]. While this may be true, our laboratory has developed a more reasonable, albeit reductionistic approach utilizing an array of a number of candidate genes that when polymorphic deviating from the wild type (normal variant) can help identify AUD persuasively [14–22].

Ten genes and 11 common polymorphisms, including Single Nucleotide Polymorphisms (SNPs) and Variable Number Tandem Repeats (VNTRs) connected to the promotion of a genetically induced hypodopaminergia met the final selection for the GARS^®^ test. The presence of hypodopaminergia is a complicated but determining condition of the GARS^®^ test results. The search for studies that report low-dopamine function associated with specific SNPs of reward genes formed the cornerstone of the development of the GARS^®^ test. While there are many possible addiction-related genes; as pointed out by Li *et al.* [23]. dopaminergic neurotransmitter pathways do not exist in isolation but rather embedded within a complex network of interrelated mesolimbic/pre-frontal Serotonergic-, Cannabinoidergic-, Endorphinergic-, GABAergic-, and Glutaminergic systems each of which exhibits a unique function within the context of addictive behaviors. Some of such interactions and polymorphisms of reward genes that have been correlated with dopamine- and reward regulation were selected to comprise the GARS^®^ genetic panel (Table 1).

Hodgkinson *et al*. [24] developed a panel of markers able to extract full haplotype information for candidate genes in alcoholism, other addictions, and disorders of mood and anxiety. A total of 130 genes were haplotyped tagged and genotyped in 7 case/control populations and 51 reference populations using Illumina Golden Gate SNP genotyping technology, determining haplotype coverage. Li *et al.* [23] who integrated 2,343 reports from peer-reviewed publications between 1976 and 2006 linking genes and chromosome regions to addiction by single-gene strategies, microarray, proteomics, or genetic studies. They identified 1,500 human addiction-related genes and developed KARG (http://karg.cbi.pku.edu.cn), the first molecular database for addiction-related genes with extensive annotations and a Web interface. They connected the common pathways into a hypothetical common molecular network for addiction including alcoholism. Interestingly, two final pathways emerged were glutamatergic dopaminergic. Moreover, research from a Brain Storm consortium quantified the genetic sharing of [25] brain disorders from genome-wide association studies of 265,218 patients and 784,643 control participants and evaluated their relationship to 17 phenotypes from 1,191,588 individuals. They found, psychiatric disorders share common variant risk, whereas neurological disorders appear more distinct from one another and from the psychiatric disorders. This dove tails with concept of Reward Deficiency Syndrome as a potential overall domain among multiple psychiatric disorders as espoused by Blum et al in 1995 [7]. These results highlight the importance of quest for additional polymorphisms as risk factors for brain disorders especially those related to psychiatric morbidity.

This is a timely and clinically relevant endeavor. For instance, alcoholism pharmacotherapy may target the brain’s reward mechanisms via the “deprivation-amplification relapse therapy” (DART) [26] Previously we proposed that low D2 receptor density and polymorphisms of the D2 gene are associated with high risk for relapse to drug consumption after some period of abstinence, that is to say craving for various substances including alcohol, heroin, cocaine, methamphetamine, nicotine, and sugar [26]. We suggested a putative physiological mechanism that may help to explain the enhanced sensitivity following intense acute dopaminergic D2 receptor activation namely, “denervation supersensitivity.” Interestingly, rodents with unilateral depletions of neo-striatal dopamine display increased sensitivity to dopamine agonists estimated to be 30 to 100 x in the 6-hydroxydopamine (6-OHDA) rotational model. Given that mild striatal dopamine D2 receptor proliferation occurs (20%-40%), it is difficult to explain the extent of behavioral supersensitivity by a simple increase in receptor density. Thus, the administration of dopamine D2 agonists would target the D2 sensitization, especially in D2 receptor A1 allele carriers and thus reduce the relapse potential. Such type of a hypothesized mechanism is supported by clinical trials utilizing amino acid neurotransmitter precursors, enkephalinase, and catechol-O-methyltransferase (COMT) enzyme inhibition, which have resulted in attenuated relapse rates in reward deficiency syndrome (RDS) probands. If future translational research reveals that dopamine agonist therapy reduces relapse in RDS, it would provide an independent support to the DART approach to DRD2 A1 allele carriers utilizing natural D2 agonist therapy by means of amino acid precursors with COMT and enkephalinase inhibition therapy [27].

RDS can be manifested in relatively mild or severe forms that follow as a consequence of an individual’s biochemical inability to derive reward from ordinary, everyday activities. DRD2 A1 Allele is indeed a very important variant as initially discovered by Blum *et al.* [28] to associate with severe alcoholism. Indeed to date there have been over five thousands PUBMED articles [9/24/20] on the DRD2 gene. Studies clearly show that striatal dopamine receptor D1-expressing neurons have been classically associated with positive reinforcement and reward, whereas D2 neurons are associated with negative reinforcement and aversion. Our laboratory has reported a series of studies and discussed the importance of D2 activation (rather than D2 blocking) as a way to improve reward processing [29]. In support of this notion, Soares-Cunha *et al.* [30], demonstrated that the pattern of activation of D1 and D2 neurons in the nucleus accumbens (NAc) predicts motivational drive, and that optogenetic activation of either neuronal population enhances motivation in mice. They further show that activating NAc D2 neurons increases cue-induced motivational drive in control animals and in a model that presents anhedonia and motivational deficits; conversely, optogenetic inhibition of D2 neurons decreases motivation. These results and known psychological deficits based even on a few dopaminergic genetic polymorphisms persuasively suggest that the classic view of D1-D2 functional antagonism as an addiction pharmacotherapy does not hold promise for various dimensions of RDS-related behaviors, and that D2 neurons play a more prominent pro-motivation role [28].

An important question relates to the importance of a multi-locus approach rather than the old adage OGOD [31] (one –gene one disease) pointing to a very complex phenotype as described by Lander & Schork [32] reviewing association studies compared to linkage analysis. Along these lines, while medical genetics was revolutionized during the 1980s by the application of genetic mapping to locate the genes responsible for simple Mendelian diseases psychiatric disorders do not follow simple inheritance patterns. Dissection of complex traits involves four major approaches: linkage analysis, allele-sharing methods, association studies, and polygenic analysis of experimental crosses. Certainly, alcoholism falls into the complex trait category.

There have been a number of studies utilizing many gene polymorphisms to access association of aberrant seeking behavior including the work of Conner *et al.*, [33] who analyzed a number of gene polymorphisms (e.g. ANKK1 ***TaqI*** A, DRD2 C957T, DRD4 7R, COMT Val/Met substitution, and SLC6A3 9R) and a GABAergic gene (GABRB3) and found hypodopaminergic functioning predicted drug use in males; however, in females, a deleterious environment was the salient predictor. This preliminary study suggests that it is possible to identify children at risk for problematic alcohol use prior to the onset of drug dependence supporting a multi locus approach but selecting appropriate alleles for each gene is required for successful identification of alcohol seeking risk behavior. Understanding that there are 30,000 genes in the human genome the exact allelic prediction seemed unattainable in the late 80s but the first clue was initiated by the seminal work of Blum & Noble [28] and subsequent binding studies [34]. Stice *et al.* [35] tested the hypotheses that humans with genotypes putatively associated with low dopamine (DA) signaling capacity, including the ***TaqIA A1*** allele, DRD2-141C Ins/Ins genotype, DRD4 7-repeat or longer allele, DAT1 9-repeat allele, and the val/met COMT genotype, and with a greater number of these genotypes per a multi-locus composite, show less responsivity of reward regions that primarily rely on DA signaling. Their findings underscore the need for polygenic testing rather than single gene approaches. Specifically, the multi-locus composite score revealed that those with a greater number of these genotypes showed less activation in reward regions, including the putamen, caudate, and insula, in response to monetary reward. The results suggest that the multi-locus genetic composite is a more sensitive index of vulnerability for low reward region responsivity than individual genotypes including the ***TaqIA A1*** allele. Similarly work from the National Institutes of Alcoholism by Tabakoff *et al.* [36] utilizing two populations of humans, implicated dopaminergic and GABA-ergic polymorphisms associated with alcohol consumption using a custom genotyping array for 1,350 single nucleotide polymorphisms.

## Summary

Genomic testing such as GARS, can improve clinical interactions and decision-making especially related to the complex trait of alcoholism [37,38]. Knowledge of precise polymorphic associations can help in the attenuation of psychological deficits and linked clinical outcomes such as guilt and denial, lack of motivation, inability to derive pleasure from normally pleasurable activities, corroboration of family gene-o-grams; assistance in risk-severity-based decisions about appropriate therapies, including pain medications and risk for addiction; choice of the appropriate level of care placement (i.e., inpatient, outpatient, intensive outpatient, residential); determination of the length of stay in treatment; determination of genetic severity-based relapse and recovery liability and vulnerability; determination of pharmacogenetic medical monitoring for better clinical outcomes (e.g., the A1 allele of the DRD2 gene reduces the binding to opioid delta receptors in the brain, thus, reducing Naltrexone’s clinical effectiveness); and supporting medical necessity for insurance scrutiny [39]. This notable paradigm shift in the face of COVID 19 seems prudent to help reduce the widespread increase in opioid deaths as suggested by Nora’s D Volkow, NIDA Director as well as elderly mistreatment [40–43]. The later becomes important when we consider the elderly and mental health issues including, anxiety, depression and even alcoholism [44] Finally, Borsook *et al.* [45], albeit, discussing pain rather than alcoholism per se provided a clear understanding concerning reward and mesolimbic functioning. They proposed the Combined Reward deficiency and Anti-reward Model (CReAM), in which biopsychosocial variables modulating brain reward, motivation and stress functions can interact in a ‘downward spiral’ fashion to exacerbate the intensity, chronicity and comorbidities of reward related endophenotypes having reduced dopaminergic function. We are therefore also proposing herein, that overcoming this potential genetic trait and even epigenetic state using metabolomic [46] principles will help RDS victims with AUD dig themselves out of the “***hypodopaminergic ditch***”

## Figures and Tables

**Table 1.** GARS® is based on the following genes linked to hypodopaminergia

Gene	Polymorphism	Location	Risk Allele	Function linked to hypodopaminergia	Reference
DRD1	rs4531 or rs4532	Chr -5	A	rs4532 is known to reduce the function of the DRD1 gene, which is needed as a “go” drive to activate D1 receptors causing normal dopamine function.	Batel *et al.* (2008) [15].
DRD2	rs1800497	Chr-11	A	rs1800497 equates functionally to 30–40% lower density of dopamine D2 receptors resulting hypodopaminergic function	Noble *et al.* (1991) [8].
DRD3	rs6280	Chr3	C	Rs6280 in D3 causes an imbalance of the DRD3 function and has associated with Heroin Dependence.	Kuo *et al* (2014) [16].
DRD4	48 base repeat VNTR	Chr 11 exon 3	Above 7 R	Similar to the D2, the VNTR 7 R and above results in a hypodopaminergia due to lower receptor function.	Van Tol (1998) [17].
COMT	rs4680	Chr 22	G	Carrying the 9 R allele leads to a high activity of catabolism of dopamine in synapse inducing a hypodopaminergia in the synapse.	Baransel Isi *et al.* (2008) [18] and Wichers *et al.* [19].
OPRM1	rs1799971	Chr 6	G	The G allele is the risk variant of the MOR 118A>G (p.Asn40Asp; SNP rs1799971) promotes a low dopamine function because there will be a lack of inhibition via the GABA inhibitory control of dopamine release at the reward site Nucleus Accumbens (NAc) inducing hypodopaminergia.	Ray *et al.* (2011) [20]
DAT1	40 base repeat VNTR	Chr 5 exon 15	9R	Carrying the 9 R allele leads to a high activity inducing a hypodopaminergia in the synapse. The DAT1 clears excess dopamine released from the pre-neuron into the synapse and prevents uptake into the receptors on the next neuron.	Byerley *et al.* (1993) [21].
MOA-A	30 base repeat VNTR	Chr X Promotor	3.5 R, 4R	The 3.5R and 4R variants are more active than 3R or 5R. Excessive amounts of dopamine are broken down in the presynaptic neuron, which may result is less dopamine availability for release into the synaptic cleft. Carriers of the 3.5 and 4R may display hypodopaminergia (low dopamine function).	Contini *et al.* (2006) [22].
5HTTLPR	43 base repeat INDEL/VNTR plus rs 25531	Chr 17	LG, S	The risk variant has 43 base –pair 5” insertion/deletion, S’ at SNP rs25531. The long allele results in higher serotonin transporter mRNA transcription in human cell lines., The result is that Serotonin is highly reabsorbed from the synapse into the prenerve cell causing low serotonin content in the synapse leading to reduced function.	Merenäkk *et al.* (2011) [23] and van der Zwaluw *et al.* (2010) [24].
GABRB3	CA repeat DNR	Chr 15 (downstream)	181	GABRA3 gene and the risk variant is CA-Repeat, whereby allele 181 results in higher activity. This risk allele, if overexpressed, will cause low dopamine function hypodopaminergia leading to SUD because of inhibition of dopamine release at the reward site.	Namkoong *et al.* (2008) [25].
